# Clinical Correlates of Cerebellar Injury in Preterm Infants with Surgical Necrotizing Enterocolitis

**DOI:** 10.21203/rs.3.rs-3720723/v1

**Published:** 2023-12-11

**Authors:** Parvesh Mohan Garg, Isabella Pittman, Charlotte Taylor, Kartik Reddy, Neha varshney, William B. Hillegass, Avinash Shetty, Joe YI, Terrie Inder, Padma Garg

**Affiliations:** Wake Forest University; University of Mississippi medical Center; University of Mississippi Medial Center; University of Mississippi Medial Center; University of Mississippi; 3. Frank Porter Graham Child Development Institute, University of North Carolina; Children’s Hospital of Orange County; University of Mississippi

## Abstract

**Objective:**

Determine the risk factors of cerebellar injury in infants with surgical necrotizing enterocolitis (NEC).

**Methods:**

Retrospective study compared clinical/pathological information between surgical NEC infants with and those without cerebellar injury.

**Results:**

Infants with cerebellar injury (21/65, 32.3%) had significantly more hemorrhagic and the reparative lesions on the intestinal histopathology, had patent ductus arteriosus (PDA) more often, received red cell transfusion frequently, had blood culture positive sepsis and grew gram positive organisms more often and had cholestasis frequently following NEC than those without cerebellar injury. On multilogistic regression, the positive blood culture sepsis (OR 3.9, CI 1.1–13.7, p = 0.03), PDA (OR 4.5, CI 1.0–19.9, p = 0.04) and severe hemorrhage (grade 3–4)(OR 16.9, CI 2.1–135.5, p = 0.007) were independently associated with higher risk of cerebellar injury.

**Conclusion:**

The cerebellar injury was most likely associated with positive blood culture sepsis following NEC, PDA, and severe hemorrhage lesions (grade 3–4) in infants with surgical NEC.

## Introduction

Necrotizing enterocolitis (NEC) is a systemic inflammatory disease of the very low birth weight infants and is associated with higher neurological morbidity, death, and increased health care cost (1–7). The surgical NEC and associated inflammation are associated with severe white matte injury lesions on the neuroimaging and adverse neurodevelopmental outcomes at two years of age (8–13). The cerebellum development processes such as proliferation and migration of neural progenitors is at high risk in preterm infants due to adverse effects associated with the preterm birth including brain hemorrhage, infection and inflammation (14).

In our recent study, we reported clinical and histopathological determinants of white matter brain injury in detail in infants with surgical necrotizing enterocolitis (NEC) and showed that MRI brain showed injury in the white matter in 52%, grey matter in 10%, and cerebellar region in 30% (15). The WMBI was most likely associated with earlier NEC onset, higher RBC transfusions, and less necrosis and greater hemorrhage lesions on intestinal pathology in preterm infants with surgical NEC(15). However, to our knowledge, the extent to which risk factors for NEC-associated cerebellar injury are not fully understood and there is no study combining clinical and postoperative course findings in identifying the subgroup of infants with surgical NEC at higher risk of cerebellar injury.

In this single-center, retrospective cohort study, we sought to determine the demographics, clinical parameters, and interventions that were associated with cerebellar injury on MRI brain at term equivalent age in preterm infants with surgical NEC.

## Methods

**Figure 1:**
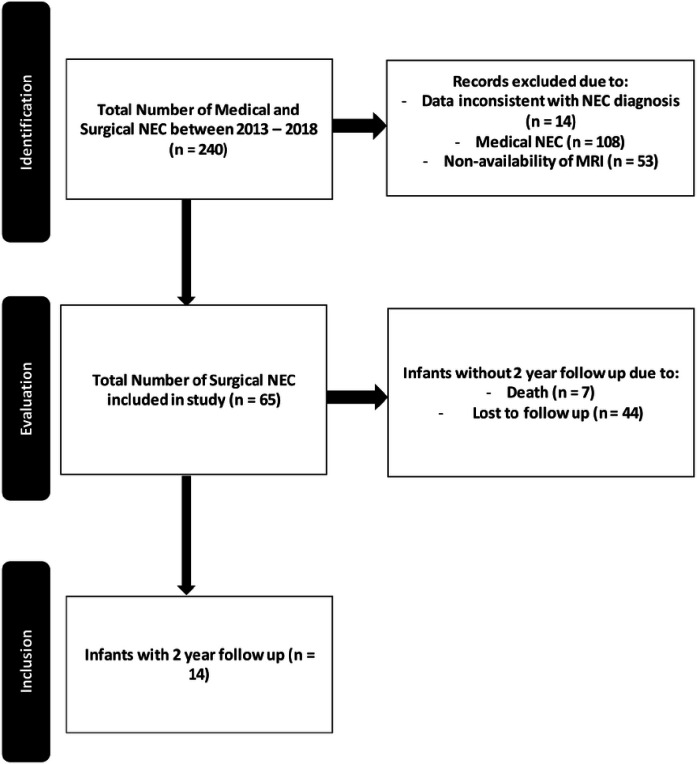
Patient flow algorithm for included, excluded, and enrolled infants with surgical necrotizing enterocolitis.

This retrospective study was conducted at the level 4 neonatal intensive care unit (NICU) at the University of Mississippi Medical Center, a regional referral center, after approval by the Institutional Review Board (2017 − 0127). A detailed review of the electronic medical records identified 243 patients with medical and surgical NEC (NEC Bell stage II and above)(16) who underwent NEC management in the period between January 2013 and December 2018. We identified 65 infants with surgical NEC qualifying for the study (see Fig. 1).

### Clinical information

We recorded demographic characteristics including birth weight, gestational age, sex, race (African American, Caucasian, or Latino), and mode of delivery (C-section / Vaginal delivery), APGAR scores at 5 minutes, out born status, and small for gestational age status. We collected information regarding maternal factors, including pregnancy-induced hypertension, chorioamnionitis, and antenatal steroids.

### NEC information

We noted the NEC features such as the age of onset and clinical presentation (abdominal distension, feeding intolerance, and bloody stools). The NEC diagnosis was made on abdominal X-ray findings such as pneumatosis, pneumoperitoneum, and portal venous gas. We recorded information on Penrose drain, time to laparotomy, length and region of bowel resected, types of stoma creation following NEC surgery.

### Histopathological Evaluation

Hematoxylin & eosin-stained surgical resected intestinal tissue sections were evaluated for necrosis, inflammation, hemorrhage, and reparative changes by a pediatric pathologist. A score of 0 was assigned when the exam appeared normal, 1 for 1–25% necrosis/ inflammation, 2 when 25–50% area involved, 3 when 50–75% area was affected, and 4 when > 75% changes were seen (17).

### Postoperative Morbidity

To assess postoperative morbidity, we recorded the duration of postoperative ileus, days of parenteral nutrition (PN) days, intestinal failure (PN > 90days), and time to achieve full feeds. Short bowel syndrome was defined as infants who were still requiring TPN at discharge or more than 90 days after NEC onset. Days of parenteral nutrition were defined as the interval between postoperative day 1 until full enteral feedings were achieved (defined as 120 ml/kg/day). Surgical morbidity was classified as surgical site infections (including dehiscence and abscesses), strictures, fistulas, adhesions, and perforations.

We recorded information on the length of stay and mortality. The length of stay was defined as the total hospitalization duration from the day of admission until discharge or death. Mortality was defined as death due to any cause prior to hospital discharge.

We also collected data on bronchopulmonary dysplasia status at 36 weeks based on the oxygen requirement at the time of assessment(18).

### Hematology information

We recorded complete blood cell count results from the electronic chart before the NEC onset (last available CBC inpatient record before NEC onset), on the day of NEC onset, 24 hours, and 48 hours after onset. We collected data on relative (presented as percentages) as well as on the absolute values. If we had multiple CBC on the same day, we recorded data from what we judged to be the most abnormal. We also collected data on platelet and RBC transfusion before and after the NEC onset.

Renal function data: We captured all serum creatinine (SCr) measurements and daily urine output (UOP) before and five days after NEC onset. After NEC onset, the incidence of AKI was determined using the modified neonatal staging criteria as previously described in the kidney disease: Improving Global Outcomes (KDIGO) Clinical Practice Guideline for AKI (19–23).

### Neonatal MRI data

All MRI brain scans (without contrast) were scored independently by two pediatric neuroradiologists unaware of the infants’ clinical course. Our NICU standard of care is to obtain a brain MRI at 36 weeks corrected age or before discharge whenever clinically feasible in neonates with birthweight less than 1500 grams. We used a standardized scoring system as used by Woodward et al. and consisting of eight 3-point scales (9) to evaluate the white matter brain injury and the gray matter brain injury.

### Cerebellar Injury

We also assessed cerebellar lesions on brain MRI. We scored the scans on a binary scale with 0 being no injury and 1 indicating the presence of cerebellar injury. Cerebellar Injury patterns that we identified on MRI brain were cerebellar hemorrhage, siderosis and/or cerebellar volume loss. Hemorrhage (superficial siderosis and parenchymal hemorrhage) detection varied between susceptibility weighted imaging (SWI) and gradient recalled echo (GRE) techniques, performed on a 1.5T or 3T strength MRI, utilizing between 1.5–2mm slices for SWI and 4–5mm slices for GRE sequences. No slice gap was used for most scans, but a more remote exam utilized a 1.5mm slice gap. Volume loss was assessed with coronal and axial T2 weighted sequences on either 1.5T or 3T strength MRI, utilizing 4–5mm slices, and no slice gap, except for two scans, which utilized fast shunt protocol technique with 2.5mm slice gap and an older MRI utilizing a 1.5mm slice gap. Two of the scans were unable to assess for hemorrhage due to motion or utilization of T2-only shunt protocol technique. Most brain MRIs were performed using the GRE sequence, which is less sensitive for the detection of hemorrhage, and a minority were performed with the more sensitive SWI technique. Asymmetric volume loss in the cerebellum was contralateral to the germinal matrix hemorrhage in all cases, a known phenomenon associated with damage to crossing white matter tracts/transsynaptic degeneration. Hemorrhages varied between location in the vermis and cerebellar hemispheres. Superficial siderosis along the cerebellum and brainstem were also noted.

### Neurodevelopment assessment at two years of age

At our center, infants underwent a neurodevelopmental comprehensive evaluation conducted by child development specialists using Bayley Scales of Infant Development (BSID-III) during the study period who were well aware of the MRI findings and the clinical course. We recorded cognitive and psychomotor development assessment scores. The Mental Development Index (MDI) assesses environmental responsiveness and sensory and perceptual abilities, memory, learning, and early language and communication abilities; the Psychomotor Development Index (PDI) assesses gross and fine motor skills.

### Statistical Methods

Normally distributed continuous variables are summarized as means and standard deviations (± SD). Comparisons between normally distributed continuous measures for those with and without cerebellar injury were performed using Student’s t-test for equal variance cases and Welch’s unequal variances t-test for unequal variances. For continuous data exhibiting non-normal distributions medians with interquartile range (IQR) [1st quartile; 3rd quartile] are presented, and differences were tested using the Kruskal-Walli’s test. Categorical data were summarized as counts with relative frequencies as percentages, and differences in the groups were analyzed using the Chi-squared test (χ² test) or Fisher’s exact test.

Univariate logistic regression analyses examined the unadjusted association between each of the risk factors and cerebellar injury. Logistic regression analyses compared clinical and pathological findings among neonates with cerebellar injury to those without cerebellar injury. Multivariate logistic regression models were used to evaluate the adjusted associations between cerebellar injury and clinical-histological factors, using absence of cerebellar injury as the reference All tests were two-sided and a p-value < 0.05 was considered statistically significant. The statistical analyses were performed in SAS 9.4 statistical software.

## Results

**Figure 2:**
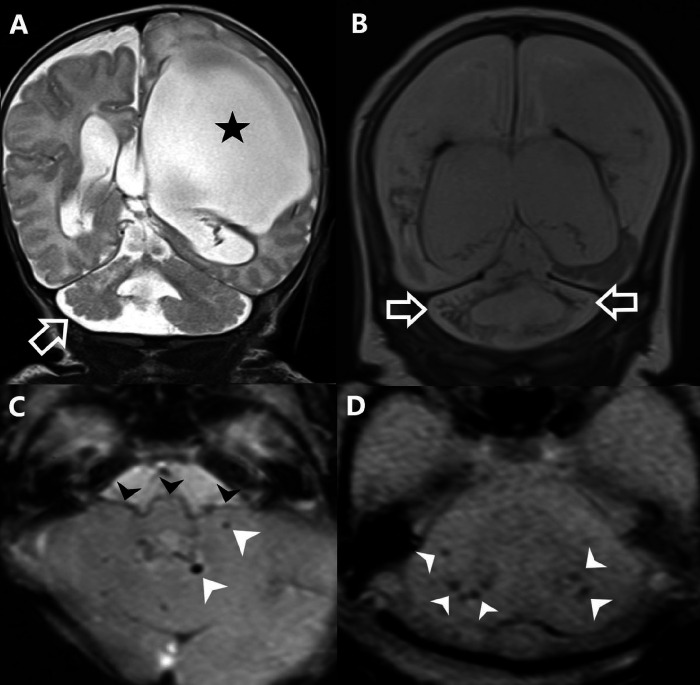
Cerebellar injury on MRI. **A**. Coronal T2 weighted MRI shows mild right cerebellar volume loss (arrow) contralateral to a large porencephalic cyst (star) in this patient with prior grade 4 germinal matrix hemorrhage. **B.** Coronal T2 MRI shows severe bilateral cerebellar volume loss (arrows) in this patient who also had a severe brain injury in the supratentorial brain. **C**. Axial SWI MRI shows few small hemorrhages in the left cerebellar hemisphere (white arrowheads) and superficial siderosis coating the surface of the brainstem and cerebellum (black arrowheads). **D**. Axial GRE shows multiple bilateral hemorrhages in the cerebellum.

Sixty-five Infants were included in the study. 21/65 (32.3%) infants had cerebellar injury. Out of 21 infants, 8/65 (12.3%) had cerebellar/superficial siderosis, 5/65 (7.6%) had cerebellar volume loss (4/5 unilateral volume loss & 1/5 bilateral volume loss) and 8/65(12.3%) had both cerebellar hemorrhages and the cerebellar volume loss. The lesions are depicted in Fig. 2.

Those with cerebellar injury had pregnancy induced hypertension less often (9.5% vs. 37.6%; p = 0.024), had less pneumatosis on the abdominal Xray (50% vs.14.3%; p = 0.006) and more hemorrhagic lesions and the reparative changes (p < 0.05) on the intestinal histopathology, had patent ductus arteriosus more often (18/21(85.7%) vs. 25/44( 56.8%);p = 0.021) received red blood cell transfusion more often (76% vs. 0%; p = 0.0001), had blood culture positive sepsis more frequently ( 13/21 ( 61.9%) vs. 11/44 ( 255);p = 0.004), grew gram positive organisms more often ( 9/21(42.9%) vs.4/44(9.1%);p = 0.001) and had cholestasis more often following NEC (18/21(85.7%) vs. 25/44 (59.5%);p = 0.035) compared to those without cerebellar injury. Preterm infants with cerebellar infants had higher mean white blood cell count (31.4 ± SD 22.9 vs. 20.9 ± SD 14.9; p = 0.080) at day 4 following NEC, higher absolute neutrophil counts at day 4 (21.5 (± SD 18.6) vs. 11.4(± SD 9.2); p = 0.023) and higher monocyte counts day 7 following NEC (14.9 ± SD6.9 vs. 21 ± SD 8.9; p = 0.022) than those without cerebellar injury on brain MRI at term equivalent age. The data are summarized in Table 1–3 and **Supplemental Table 1**.

Those with cerebellar injury more likely had higher associated white matter injury (19/21 (90.5%) vs. 15/44(34.1%); p = 0.0001) and higher grade 3–4 WMI (14/21 (66.7%) vs. 4/44(9.1%) p = 0.0005) and higher ROP on eye exam (70.6% vs. 38.5%; p = 0.027) than those without cerebellar injury.

### Two-year Neurodevelopmental Outcomes

There were no significant differences in the cognitive, language, motor and the socio-emotional scores assessed by BSID III at 2 years of corrected age in surgical NEC infants with and without cerebellar injury. The data are summarized in the Table 3.

### Multiregression Analysis

On multi logistic regression analysis the positive blood culture sepsis (OR 3.9, CI 1.1–13.7, p = 0.0368), patent ductus arteriosus (OR 4.5 (1.0–19.9, p = 0.047) and the severe hemorrhage (grade 3–4) (OR 16.9, CI 2.1–135.5, p = 0.0079) were independently associated with higher risk of cerebellar injury on the brain MRI at term equivalent age. Table 4

## Discussion

Our study has demonstrated that the cerebellar injury in 32% of cases on TEA MRI brain in preterm infants with surgical NEC. Infants with cerebellar injury were less likely associated with pregnancy induced hypertension less often, had less pneumatosis, had patent ductus arteriosus more often and had more hemorrhagic lesions and the reparative changes on the intestinal histopathology. Those with cerebellar injury received red blood cell transfusion more often, had blood culture positive sepsis more frequently, grew gram positive organisms more often and had cholestasis more often following NEC compared to those without cerebellar injury on the univariate analysis. However, the positive blood culture sepsis, patent ductus arteriosus and the severe hemorrhage (grade 3–4) remained independently associated with higher risk of cerebellar injury on the brain MRI at term equivalent age on the multilogistic regression analysis.

Our study noted a higher gram-positive infection frequency than infants with cerebellar injury. *S. epidermidis* sepsis was associated with higher odds for neurodevelopmental impairment (OR 1.31, 95% CI: 1.09–1.57) compared to control in a recent meta-analysis (24). Neonatal host response to *S. epidermidis* sepsis has not been fully understood. It is most likely due to immature innate immunity with a distinctive regulation pattern of the inflammatory response (25). A prospective study of 192 neonates (gestational age < 30 weeks) noted that infants with gram-positive infection had significantly more white matter injury on the brain MRI than those with no sepsis-associated NEC (13). Bacteremia-induced brain injury may be explained by the release of lipopolysaccharide or peptidoglycan and modulating pro-inflammatory genes in the brain such as Toll-like receptors, nuclear factor-κB, antioxidants, oxidants, and cytokines (26).

Geier et al has shown that patients with cholestasis (direct bilirubin > 2 mg/dl) had a higher incidence of bloodstream infections following surgical NEC; in sepsis-associated liver injury, bacterial toxins may have induced pro-inflammatory cytokines and caused ischemic liver injury (27). In our cohort infants with cerebellar injury had higher evidence of pneumatosis on the abdominal x-rays. Pneumatosis in NEC infants is due to establishment of the gas-producing bacteria by 3–4 weeks of life and translocation of gas-producing bacteria to the subserosa layer. La Rosa et al. have shown that the gut microbiota of premature infants residing in a tightly controlled microbial environment progresses through a choreographed succession of bacterial classes from Bacilli to Gammaproteobacteria to Clostridia and slow progression in infants with the lower gestational age (28).

In this cohort, infants with cerebellar injury had more hemorrhagic lesions on the intestinal pathology and received more packed red cell transfusion. In our study, infants with WMBI received more red blood transfusions before NEC onset than the group without WMBI [n = 14 (41.2%) vs. n = 2(9.1%); p = 0.009]. A recent study reported the impact of blood transfusions on neurodevelopmental outcomes in the Preterm Erythropoietin (Epo) Neuroprotection (PENUT) Trial population. Each transfusion was associated with a decrease in mean cognitive score of 0.96 (95% CI [1.34, 0.57]), a decrease in mean motor score of 1.51 [−1.91, −1.12], and a decrease in mean language score of 1.10 [−1.54, −0.66](29). The exact mechanism of brain injury remains unclear, but possible mechanisms include pro-inflammatory injury, suppression of endogenous erythropoietin, and oxidative stress mediating injury to the pre-oligodendroglia following blood transfusion (30).

Animal studies have reported systemic inflammation secondary to NEC leading to neuronal injury via microglial activation, inflammatory pathway activation, and brain barrier disruption (31–34). A study done in non-primate baboon model has shown that preterm birth followed by neonatal intensive care experience for 2 weeks impeded the Purkinje cells including action potential waveforms, synaptic input, and dendritic extension compared with age matched controls (35).Cha et al. has shown that altered white matter microstructure in preterm infants with and without NEC. They reported significantly increased mean diffusivity in the splenium of corpus callosum (p = 0.001) and the left corticospinal tract (p = 0.001) in preterm infants with NEC (36). Jiang et al reported that neonatal NEC adversely affects myelination of the more rostral or central regions of the immature brainstem as evidenced by the maximum length sequence brainstem auditory evoked response components, resulting in delayed or impaired neural conduction, but spares the more peripheral regions (37).

This study’s strengths include that it is one of the few studies to identify clinical and pathological risk factors for the cerebellar injury in preterm infants with surgical necrotizing enterocolitis. Identification of risk factors associated with cerebellar injury may improve early recognition of at-risk preterm infants and provide useful bedside prognostic information. Limitations of our study include that it is single-center experience and retrospective. The relatively small sample size may reduce the study’s generalizability and the statistical power to detect additional important associations between clinical determinants and cerebellar injury in a neonate with surgical necrotizing enterocolitis. Secondly, we agree that the number of comparisons given our cohort size generates high probability of type I errors. Thirdly, we did not see any significant neurodevelopmental outcomes at 2 years of corrected age most likely due to poor patient follow up rate.

In conclusion, this study demonstrates that cerebellar injury was seen in 32% of infants with surgical necrotizing enterocolitis. Those with cerebellar injury received red blood cell transfusion frequently, had severe hemorrhagic lesions on the intestinal histopathology, had blood culture positive sepsis at the time of NEC onset more frequently, grew gram positive organisms on the blood culture more often and had cholestasis more frequently following NEC compared to those without cerebellar injury.

In the future, prospective multi-center studies, which allow the inclusion of additional clinical details (e.g., gut perfusion, gut microbiome) and laboratory predictors such as inflammatory biomarkers, may support earlier recognition of cerebellar injury or identify other risk factors cerebellar injury following surgical NEC. In the NEC setting some of these exposures are non-modifiable or unavoidable, however this highlights the value of assessing clinical/pathological risk factors in infants diagnosed with NEC, given the higher risk cerebellar injury. Studies that evaluate neuroprotective strategies to prevent cerebellar injury, and consequences are greatly needed to improve neurodevelopmental outcomes in high-risk preterm infants with NEC. Our findings may provide further guidance in targeting experimental neuroprotective or mitigating interventions.

## Figures and Tables

**Table 1:** Demographics and the clinical information in infants with and without cerebellar Injury

	N	Total (N = 65)	Cerebellar Injury (N = 21)	No Cerebellar Injury (N = 44)	P Value
Prenatal Information					
Chronic Hypertension, n (%)	59	10 (16.9)	0 (0.0)	10 (25.6)	0.013
Antenatal Steroid Use, n (%)	64	49 (76.6)	17 (81.0)	32 (74.4)	0.56
Pregnancy-Induced Hypertension, n (%)	65	18 (27.7)	2 (9.5)	16 (36.4)	0.024
Chorioamnionitis, n (%)	65	8 (12.3)	4 (19.0)	4 (9.1)	0.25
Clinical information					
Gestational age (weeks, mean ± SD)	65	26.4 (2.7)	26.3 (2.7)	26.5 (2.7)	0.88
Birth weight (g, mean ± SD)	65	923.9 (495.3)	928.4 (457.4)	921.7 (517.5)	0.96
Male, n (%)	65	43 (66.2)	15 (71.4)	28 (63.6)	0.54
Ethnicity, n (%)	65				0.74
African American		12 (18.5)	4 (19.0)	8 (18.2)	
Caucasian		49 (75.4)	16 (76.2)	33 (75.0)	
Latino		2 (3.1)	0 (0.0)	2 (4.5)	
Other		2 (3.1)	1 (4.8)	2 (2.3)	
Small for gestational age, n (%)	64	17 (26.6)	5 (25.0)	12 (27.3)	0.85
Out born, n (%)	65	39 (60.0)	15 (71.4)	24 (54.5)	0.19
Assisted ventilation (intubated), n (%)	55	47 (85.5)	18 (94.7)	29 (80.6)	0.33
Pressor support 24 h after NEC, n (%)	64	50 (78.1)	19 (90.5)	31 (72.1)	0.10
Indomethacin use, n (%)	65	9 (13.8)	5 (23.8)	4 (9.1)	0.11
Platelet transfusion before NEC, n (%)	65	45 (69.2)	15 (71.4)	30 (68.2)	0.79
PRBC transfusion before NEC, n (%)	56	16 (28.6)	16 (76.2)	0 (0.0)	0.000
PDA, n (%)	65	43 (66.2)	18 (85.7)	25 (56.8)	0.021
BPD, n (%)	57	50 (87.7)	17 (100.0)	33 (82.5))	0.07
Severe BPD, n (%)	56	43 (76.8)	16 (84.2)	27 (73.0)	0.35
Home O2 use, n (%)	39	13 (33.3)	7 (58.3)	6 (22.2)	0.027
Postnatal steroid use, n (%)	65	40 (61.5)	14 (66.7)	26 (59.1)	0.56
Type of steroid used n (%)	34				0.14
Hydrocortisone		30 (88.2)	11 (100.0)	19 (82.6)	
Dexamethasone		4 (11.8)	0 (0.0)	4 (17.4)	
Any AKI present following NEC, n (%)	57	40 (70.2)	16 (84.2)	24 (63.2)	0.10
AKI by serum creatinine, n (%)	57	30 (52.6)	13 (68.4)	17 (44.7)	0.09
AKI by urine output, n (%)	57	23 (40.4)	5 (26.3)	18 (47.4)	0.13
AKI by urine output and serum creatinine present, n (%)	58	12 (20.7)	5 (25.0)	7 (18.4)	0.56
Discharge					
Length of stay (days, mean ± SD)	65	160.88 (75.7)	169.3 (70.0)	156.9 (78.7)	0.54
Death	65	5 (7.7)	3 (14.3)	2 (4.5)	0.17

NEC necrotizing enterocolitis. PDA patent ductus arteriosus. SGA small for gestational age. BPD bronchopulmonary dysplasia. AKI = Acute Kidney injury. Categorical variables are presented as count (percentage) and continuous variables are presented as mean (standard deviation). Differences in continuous measures were tested using a t test, ANOVA, or Kruskal-Walli’s test. Differences in categorical measures were tested using the χ2 test. The presence of bold and italic values signified p < 0.05.

**Table 2:** NEC information and the post-operative course in infants with and without cerebellar Injury

	N	Total (N = 65)	Cerebellar Injury (N = 21)	No Cerebellar Injury (N = 44)	P Value
NEC Disease Features					
NEC age of onset (days, mean ± SD)	65	17.4 (15.8)	12.6 (13.8)	19.7 (16.4)	0.09
Radiologic Findings, n (%)	65				
Pneumatosis		25 (38.5)	3 (14.3)	22 (50.0)	0.006
Portal venous gas		3 (4.6)	0 (0.0)	3 (6.8)	0.22
Pneumoperitoneum		36 (55.4)	15 (71.4)	21 (47.7)	0.07
Necrosis, n (%)	57				0.09
0%		15 (26.3)	5 (25.0)	10 (27.0)	
< 25%		9 (15.8)	6 (30.0)	3 (8.1)	
25–50%		14 (24.6)	6 (30.0)	8 (21.6)	
50–75%		14 (24.6)	3 (15.0)	11 (29.7)	
> 75%		5 (8.8)	0 (0.0)	5 (13.5)	
Inflammation, n (%)	57				0.34
0%		3 (5.3)	1 (5.0)	2 (5.4)	
< 25%		18 (31.6)	5 (25.0)	13 (35.1)	
25–50%		31 (54.4)	14 (70.0)	17 (45.9)	
50–75%		1 (1.8)	0 (0.0)	1 (2.7)	
> 75%		4 (7.0)	0 (0.0)	4 (7.0)	
Hemorrhage, n (%)	54				0.000
0%		3 (5.6)	0 (0.0)	3 (8.8)	
< 25%		10 (18.5)	2 (10.0)	8 (23.5)	
25–50%		21 (38.9)	3 (15.0)	18 (52.9)	
50–75%		7 (13.0)	3 (15.0)	4 (11.8)	
> 75%		13 (24.1)	12 (60.0)	1 (2.9)	
Reparative change, n (%)	53				0.001
0%		31 (58.5)	10 (50.0)	21 (63.6)	
< 25%		14 (26.4)	2 (10.0)	12 (36.4)	
25–50%		6 (11.3)	6 (30.0)	0 (0.0)	
50–75%		2 (3.8)	2 (10.0)	0 (0.0)	
Length of Bowel resected (cm, mean ± SD)	57	18.5 (20.4)	16.7 (16.2)	19.4 (22.4)	0.64
Penrose drain, n (%)	61	25 (41.0)	7 (33.3)	18 (45.0)	0.38
Presence of ileocecal valve, n (%)	64	48 (75.0)	15 (71.4)	33 (76.7)	0.65
Laparotomy at < 48 hours, n (%)	60	42 (70.0)	16 (76.2)	26 (66.7)	0.44
Laparotomy at > 48 hours, n (%)	60	18 (30.0)	4 (20.0)	14 (35.0)	0.23
Jejunostomy, n (%)	65	21 (32.3)	6 (28.6)	15 (34.1)	0.66
Ileostomy, n (%)	65	36 (55.4)	15 (71.4)	21 (47.7)	0.07
Colostomy, n (%)	65	3 (4.6)	0 (0.0)	3 (6.8)	0.22
Region of bowel resected, n (%)	60				0.75
Small Bowel		40 (66.7)	14 (70.0)	26 (65.0)	
Large bowel		1 (1.7)	0 (0.0)	1 (2.5)	
Combined Large and Small Bowel		19 (31.7)	6 (30.0)	13 (32.5)	
Sepsis Variables					
Positive blood culture sepsis, n (%)	65	24 (36.9)	13 (61.9)	11 (25.0)	0.004
Cholestasis, n (%)	63	43 (68.3)	18 (85.7)	25 (59.5)	0.035
Gram-positive sepsis, n (%)	65	13 (20.0)	9 (42.9)	4 (9.1)	0.001
Gram-negative sepsis, n (%)	65	8 (12.3)	3 (14.3)	5 (11.4)	0.74
CRP on the day of NEC (mg/dL, mean ± SD)	56	7.4 (8.9)	6.8 (8.2)	7.7 (9.2)	0.73
CRP 24 hours after NEC (mg/dL, mean ± SD)	49	12.5 (12.2)	12.4 (12.2)	12.5 (12.4)	0.97
CRP 48 hours after NEC (mg/dL, mean ± SD)	44	12.2 (10.4)	12.4 (12.2)	12.5 (12.4)	0.96
CRP 96 hours after NEC (mg/dL, mean ± SD)	46	9.6 (9.3)	9.9 (12.8)	9.4 (7.0)	0.86
CRP 1 week after NEC (mg/dL, mean ± SD)	45	8.0 (9.5)	8.5 (12.6)	7.8 (8.0)	0.81
CRP 2 weeks after NEC (mg/dL, mean ± SD)	48	3.9 (3.8)	3.8 (3.4)	4.0 (4.1)	0.87
Central line days (days, mean ± SD)	63	63.2 (41.3)	76.0 (50.4)	56.8 (34.8)	0.08
Post-operative Intestinal Features					
Time to reach full feeds (days, mean ± SD)	58	66.4 (40.3)	77.1 (51.0)	61.3 (33.5)	0.16
Days of starting feeds (days, mean ± SD)	62	17.9 (14.9)	16.7 (7.5)	18.5 (17.4)	0.65
Achievement of full feeds, n (%)	54	48 (88.9)	16 (84.2)	32 (91.4)	0.42
Days of PN (days, mean ± SD)	65	105.5 (58.9)	118.5 (62.0)	99.3 (57.1)	0.22
Post-operative ileus (days, mean ± SD)	63	15.8 (12.4)	16.0 (7.5)	15.7 (14.1)	0.94
Surgical Complication, n (%)	65	26 (40.0)	8 (38.1)	18 (40.9)	0.83
Intestinal Failure, n (%)	58	26 (44.8)	8 (44.4)	18 (45.0)	0.97
Cholestasis, n (%)	63	43 (68.3)	18 (85.7)	25 (59.5)	0.035

NEC = necrotizing enterocolitis. CRP = C-reactive protein. PN = Parenteral nutrition. Categorical variables are presented as count (percentage) and continuous variables are presented as mean (standard deviation). Differences in continuous measures were tested using a t test, ANOVA, or Kruskal-Wallis test. Differences in categorical measures were tested using the χ2 test. The presence of bold and italic values signified p < 0.05.

**Table 3:** Neurodevelopmental outcomes in infants with and without cerebellar Injury

	N	Total (N = 65)	Cerebellar Injury (N = 21)	No Cerebellar Injury (N = 44)	P Value
**White matter brain injury lesions**					
Any WMI Present, *n* (%)	65	34 (52.3)	19 (90.5)	15 (34.1)	**0.0001**
Severe WMI (score 3–4), *n* (%)	65	18 (27.7)	14 (66.7)	4 (9.1)	**0.0001**
**Neurodevelopmental Outcomes**					
Language scores (mean ± SD)	14	66.2 (11.2)	62.0 (18.7)	67.4 (9.2)	0.48
Cognitive scores (mean ± SD)	14	72.7 (12.1)	65.0 (14.1)	75.0 (11.0)	0.16
Motor scores (mean ± SD)	14	71.2 (15.0)	59.5 (11.1)	74.9 (14.4)	0.07
Social/emotional scores (mean ± SD)	14	84.3 (17.4)	83.3 (20.2)	84.6 (17.7)	0.92
ROP *n* (%)	56	27 (48.2)	12 (70.6)	15 (38.5)	**0.027**
Hearing loss, *n* (%)	65	7 (10.8)	2 (9.5)	5 (11.4)	**0.823**

NEC necrotizing enterocolitis. WMI white matter injury. ROP retinopathy of prematurity. Categorical variables are presented as count (percentage) and continuous variables are presented as mean (standard deviation). Differences in continuous measures were tested using a t test, ANOVA, or Kruskal-Walli’s test. Differences in categorical measures were tested using the χ2 test. The presence of bold and italic values signified p < 0.05.

**Table 4.** Associations between Cerebral Injury and candidate covariates

N=59	Exp (B)	95% CI	Significance
Cholestasis	2.2	0.5 – 10.0	0.3015
Patent Ductus Arteriosus	4.5	1.0 – 19.9	0.0477
Positive Blood Culture Sepsis	3.9	1.1 – 13.7	0.0368
Blood Transfusion	low sample size due to one group with zero value.		.
Hemorrhage	16.9	2.1 – 135.5	0.0079
Reparative Change	3.8	0.5 – 30.9	0.2127
Pneumatosis	0.1	0.01 – 1.13	0.0633
PIH	0.28	0.02 – 3.64	0.3281

## Impact

1. In preterm infants with surgical NEC, brain magnetic resonance injury (MRI) showed cerebellar region in 30%.
2. Preterm infants with cerebellar injury (grade 3–4) had greater hemorrhagic lesions on histopathology of the bowel.
3. Preterm infants with cerebellar injury had positive blood culture sepsis and most likely associated with gram positive bacteremia.
4. Neuroprotective strategies to prevent cerebellar injury in preterm infants with surgical NEC are needed with the goal of improving the neurodevelopmental outcomes.
